# Carbon dots for the treatment of cancer-related anemia

**DOI:** 10.1097/BS9.0000000000000120

**Published:** 2022-07-01

**Authors:** Xu Han, Peng Ji

**Affiliations:** a Department of Pathology, Feinberg School of Medicine, Northwestern University, Chicago, IL; b Robert H. Lurie Comprehensive Cancer Center, Northwestern University, Chicago, IL

Erythropoiesis is the process in which hematopoietic stem cells (HSCs) differentiate, proliferate, and eventually form mature red blood cells.^1^ The early phase of erythropoiesis involves HSC commitment to erythroid progenitors including burst-forming unit-erythroid (BFU-E) and colony forming unit-erythroid (CFU-E). This is followed by terminal erythropoiesis in which morphologically recognizable erythroblasts proliferate and differentiate to enucleated red blood cells.^2,3^ Each of these steps is coordinated by a highly complex regulatory network. Disruption of any component of this network leads to a variety of erythroid-related disorders, manifested mainly as anemia.^4^

Anemia is a major public health problem worldwide.^5^ While primary anemia is usually caused by abnormalities in the hematopoietic system, secondary anemia can be induced by chronic diseases such as cancers.^6–9^ Cancer-related anemia is a common condition with prevalence of 30% to 90% depending on cancer types and the disease stage.^10^ Anemia significantly reduces the quality of life and shortens survival in cancer patients.^8^

The most common treatment options for cancer-related anemia include iron supplements, red cell transfusion, and erythropoietic-stimulating agents (ESAs) including erythropoietin (EPO) and its analogs. As an important cytokine for erythropoiesis, EPO is indispensable for erythropoiesis in proliferation, survival, and maturation; thus, it is commonly used for treating cancer-related anemia.^11^ EPO and ESAs were believed to be safe and effective in these patients. However, studies in the past decade raised concerns on potential effects of EPO in promoting tumor cell proliferation and metastasis.^7^ Therefore, the development of new agents or therapies that are effective and safe is an unmet medical need to treat cancer-related anemia.

**Figure 1.**
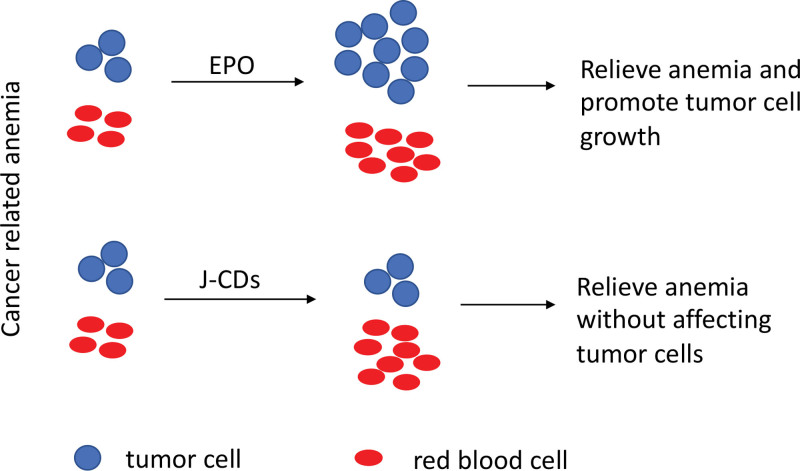
Comparing EPO and J-CDs to treat cancer-related anemia: EPO treatment increases proliferation of both red blood cells and tumor cells, leading to relieved anemia but shortened survival. J-CDs treatment increases red blood cell production without affecting tumor cells, leading to relieved anemia and normal survival. EPO = erythropoietin, J-CDs = Jujube-derived carbon dots.

Recently, studies from Xu et al shed light on solutions to this unmet need by showing carbon dots (CDs), as a novel class of agents, to be able to promote red blood cell production and efficiently treat animal models of cancer-related anemia.^12^ In their study, distinctive biological CDs, derived from red dates (Jujube-derived CDs [J-CDs]), were synthesized. Further analyses showed that J-CDs had biocompatibility and membrane permeability that are suitable for the tests on their roles in erythropoiesis. To this end, J-CDs were first tested in an in vitro erythroid-differentiation system and found to be able to induce red blood cell production via promoting the self-renewal of BFU-E and CFU-E without affecting terminal erythropoiesis. This finding was confirmed by an in vivo assay. J-CDs treatment increases mouse bone marrow and spleen erythropoiesis similarly through promoting BFU-E and CFU-E self-renewal. Hematopoietic stem and progenitor cell (HSPC) populations and nonerythroid lineages were not affected by J-CDs. Importantly, J-CDs were compared with EPO in several cancer cell lines and a NOD/SCID xenograft tumor model. EPO treatment promoted proliferation of both red blood cells and cancer cell lines, leading to shortened survival in mice with grafted tumor cells. However, J-CDs only led to red blood cell production without induction of tumor growth or affecting animal survival (Fig. 1). The authors also explored a possible mechanism of function of J-CDs by showing that J-CDs may regulate the hypoxia response and increase STAT5 phosphorylation and ultimately promote the self-renewal of erythroid progenitor cells. Taken together, J-CDs were shown to be promising agents for treating cancer-related anemia.

Interdisciplinary studies in drug development often provide more therapeutic choices. As new nanocomposite materials with suitable biocompatibility and specific physicochemical characteristics, CDs were proved to have therapeutic efficiency in Parkinson’s disease and cancer.^13,14^ In the studies from Xu et al,^12^ J-CDs were used to treat anemia since red dates are considered to be “blood tonic” in traditional Chinese medicine. An interesting finding is that red dates/jujube powders did not promote red blood cell production. The powders also failed to show any effect on the erythroid progenitor cells, indicating that J-CDs had unique properties and roles compared with red dates/jujubes. The finding that J-CDs only promote self-renewal of BFU-E and CFU-E without affecting other hematopoietic populations also indicates that J-CDs may have erythroid progenitor-specific mechanisms, which warrants detailed future investigation. Further studies including exploring the direct binding targets of J-CDs could provide a comprehensive understanding on their specificity to erythroid progenitor cells. Another major question is whether CDs in general, that is, not specifically derived from red dates, could also play similar functions in promoting red blood cell production.

The work from Xu et al successfully applied nanocomposite material in the biomedicine field. The development of J-CDs not only provides a new and promising choice to treat cancer-related anemia but also widens the application of nanocomposite materials. In addition, this study has important social and economic values in promoting the modernization of traditional Chinese medicine.
